# Synthetic T2-weighted fat sat based on a generative adversarial network shows potential for scan time reduction in spine imaging in a multicenter test dataset

**DOI:** 10.1007/s00330-023-09512-4

**Published:** 2023-03-16

**Authors:** Sarah Schlaeger, Katharina Drummer, Malek El Husseini, Florian Kofler, Nico Sollmann, Severin Schramm, Claus Zimmer, Benedikt Wiestler, Jan S. Kirschke

**Affiliations:** 1 Department of Diagnostic and Interventional Neuroradiology, School of Medicine, Klinikum rechts der Isar, Technical University of Munich, Munich, Germany; 2 Department of Informatics, Technical University of Munich, Munich, Germany; 3 TranslaTUM - Central Institute for Translational Cancer Research, Technical University of Munich, Munich, Germany; 4 Helmholtz AI, Helmholtz Zentrum München, Munich, Germany; 5 TUM-NeuroImaging Center, Klinikum rechts der Isar, Technical University of Munich, Munich, Germany; 6 Department of Diagnostic and Interventional Radiology, University Hospital Ulm, Ulm, Germany

**Keywords:** Magnetic resonance imaging, Spine, Artificial intelligence

## Abstract

**Objectives:**

T2-weighted (w) fat sat (fs) sequences, which are important in spine MRI, require a significant amount of scan time. Generative adversarial networks (GANs) can generate synthetic T2-w fs images. We evaluated the potential of synthetic T2-w fs images by comparing them to their true counterpart regarding image and fat saturation quality, and diagnostic agreement in a heterogenous, multicenter dataset.

**Methods:**

A GAN was used to synthesize T2-w fs from T1- and non-fs T2-w. The training dataset comprised scans of 73 patients from two scanners, and the test dataset, scans of 101 patients from 38 multicenter scanners. Apparent signal- and contrast-to-noise ratios (aSNR/aCNR) were measured in true and synthetic T2-w fs. Two neuroradiologists graded image (5-point scale) and fat saturation quality (3-point scale). To evaluate whether the T2-w fs images are indistinguishable, a Turing test was performed by eleven neuroradiologists. Six pathologies were graded on the synthetic protocol (with synthetic T2-w fs) and the original protocol (with true T2-w fs) by the two neuroradiologists.

**Results:**

aSNR and aCNR were not significantly different between the synthetic and true T2-w fs images. Subjective image quality was graded higher for synthetic T2-w fs (*p* = 0.023). In the Turing test, synthetic and true T2-w fs could not be distinguished from each other. The intermethod agreement between synthetic and original protocol ranged from substantial to almost perfect agreement for the evaluated pathologies.

**Discussion:**

The synthetic T2-w fs might replace a physical T2-w fs. Our approach validated on a challenging, multicenter dataset is highly generalizable and allows for shorter scan protocols.

**Key Points:**

• *Generative adversarial networks can be used to generate synthetic T2-weighted fat sat images from T1- and non-fat sat T2-weighted images of the spine.*

• *The synthetic T2-weighted fat sat images might replace a physically acquired T2-weighted fat sat showing a better image quality and excellent diagnostic agreement with the true T2-weighted fat images.*

• *The present approach validated on a challenging, multicenter dataset is highly generalizable and allows for significantly shorter scan protocols.*

**Supplementary Information:**

The online version contains supplementary material available at 10.1007/s00330-023-09512-4.

## Introduction

Magnetic resonance imagining (MRI) plays an outstanding role in the assessment of spine pathologies due to its high soft tissue contrast, its non-invasiveness, the lack of radiation exposure, and the possibility for a multiparametric image acquisition [1, 2].

Routinely, sagittal T1-weighted (w) sequences (− / + contrast agent) and T2-w sequences are acquired [2]. Additionally, sagittal T2-w sequences combined with fat suppression or separation techniques have become an important part of spine imaging [2]. The removal of the contribution of the fat signal to the overall MR signal enhances contrast resolution, improves assessment of pathologies characterized by changes of the fluid concentration, reduces artifacts, and facilitates the decision of whether additional contrast agent is needed [2–14]. Particularly for the diagnosis of acute pathologies such as inflammation or acute vertebral fractures, T2-w fs images are essential [15].

However, acquiring an additional T2-w fat sat (fs) sequence requires longer scan protocols, which decreases the MR throughput [16]. Prolonged acquisition times reduce patient comfort which could contribute to motion artifacts in imaging data. Additionally, spectral fat saturation techniques are particularly prone to artifacts caused by field inhomogeneities, e.g., around metal implants [4].

Parallel to advancement of MRI acceleration techniques [17, 18], recently, virtually generated MR images offer a promising approach for scan time reduction, as the physical acquisition of particular sequences is no longer necessary. Generative adversarial networks (GANs) based on a deep-learning (DL) architecture can be used to generate such synthetic images from different MR contrasts as input [19–23]. The iterative interaction of two networks, one generating images and one learning to differentiate between synthetic and true images [24, 25], has already been used on MRI data from a variety of anatomical regions [26–28]. In the spine, GANs can generate T2-fs images from conventional T1-w and non-fs T2-w images [15, 29]. Thereby, apart from scan time acceleration, the synthetic T2-w fs images might be less prone to artifacts, as the synthetic images are based on technically stable T1-w and non-fs T2-w images as input.

To foster a widespread implementation of GAN-based T2-w fs images in research and clinical spine imaging, synthetic images need to pass a validation by radiologists’ perception and the GAN framework has to prove external validity.

Hence, our work aims to investigate the diagnostic performance of a sagittal, GAN-based T2-w fs of the spine generated from heterogenous, multicenter T1-w and T2-w images. We hypothesized that synthetic T2-w fs images represent a good alternative to true T2-w fs images consequently allowing shorter scan protocols. Therefore, synthetic T2-w fs images were compared to their true counterparts regarding (1) image quality (quantitatively, qualitatively and with a visual Turing test) and fs quality (qualitatively) and (2) diagnostic agreement (qualitatively).

## Methods

### Magnetic resonance imaging data

#### Subject population

We retrospectively identified 201 patients with sagittal T1-w turbo spin echo (TSE), T2-w TSE, and T2-w TSE fs images of the spine. The study design was approved by the local ethics commission. Informed consent was waived due to the retrospective character.

#### Training data

**Fig. 1:**
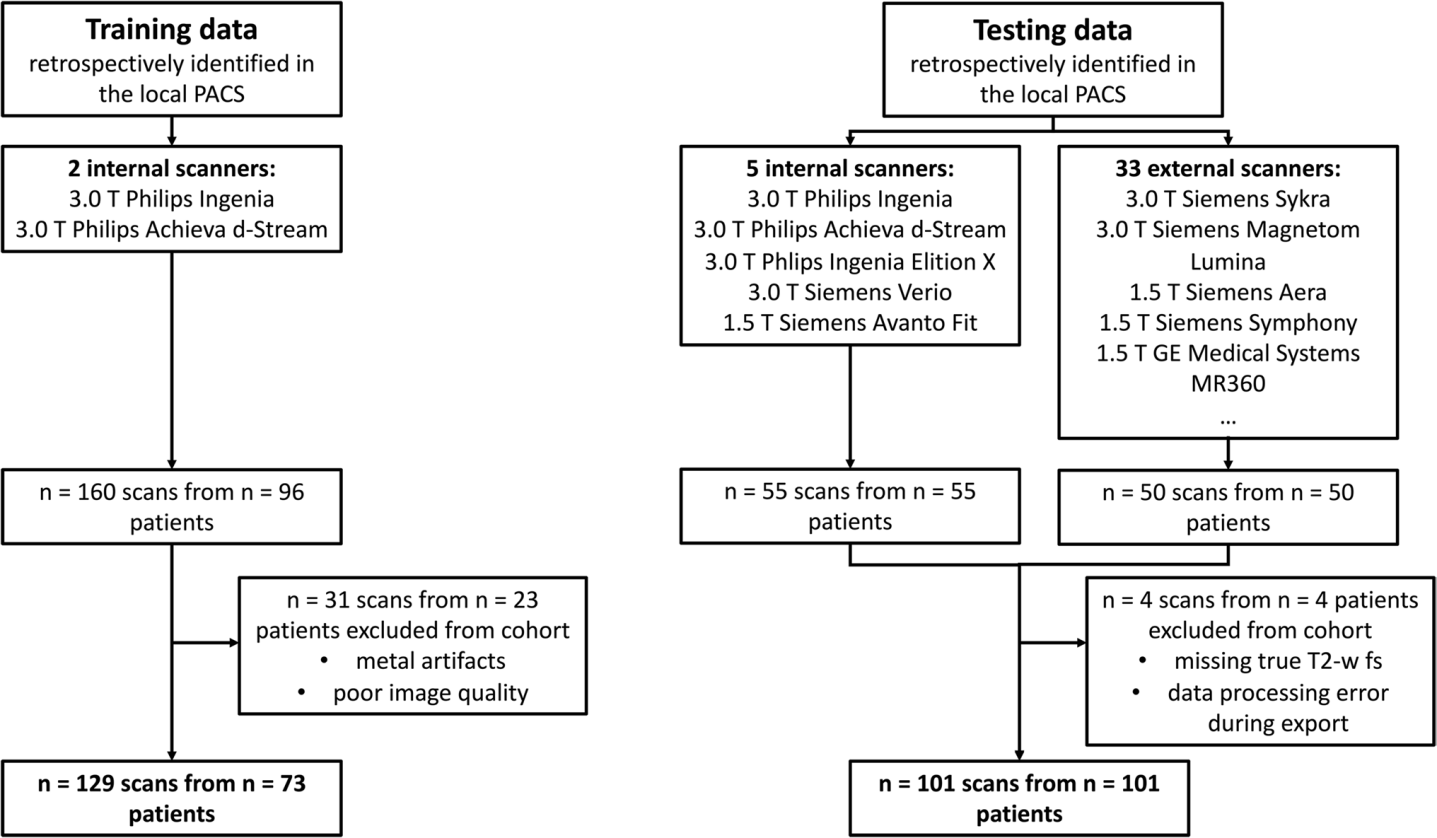
Flow chart describing inclusion and exclusion criteria of training and testing data

Training data for the GAN was retrospectively retrieved from 160 sagittal T1-w, T2-w, and T2-w fs spine images of 96 patients. Due to metal artifacts or poor image quality, 31 scans were excluded (only in the training data) (Fig. 1). All scans originated from two in-house 3 T scanners (Ingenia and Achieva d-stream, Philips Healthcare) using a similar protocol. Sequence parameters are given in Table SM1.

#### Testing data

**Fig. 2:**
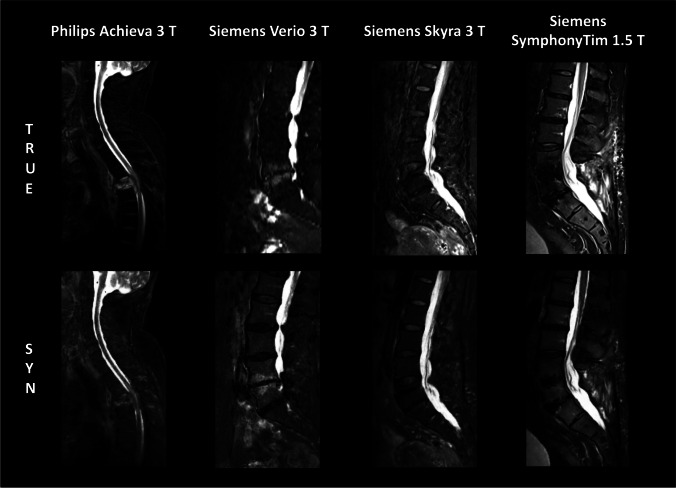
Exemplary images of true and synthetic T2-w fs from different scanner hardware

We retrospectively identified 105 MRI datasets of 105 patients consisting of sagittal T1-w, T2-w, and T2-w fs scans. Starting with date 2020/10/01 and going backward, all subsequent spine scans uploaded to the PACS were included up to a number of 105 datasets. Thereby, in-house scans (*n* = 55) and scans from other institutions (*n* = 50) being imported for clinical review were included. Four datasets were excluded due to missing true T2-w fs images or data processing errors during export (Fig. 1). Notably, artifacts, e.g., due to foreign material or poor image quality, did not represent an exclusion criterion to assess the performance of the GAN also in these challenging situations. The remaining 101 datasets originated from *n* = 38 scanners from three vendors (Philips Healthcare; Siemens Healthineers; GE Healthcare). Figure 2 shows images of true and synthetic T2-w fs from different scanner hardware. *n* = 41 datasets were acquired at 1.5 T, *n* = 60 datasets at 3 T. Slice thickness ranked from 2.2 to 5.5 mm; field of view (FOV) *x*/*y*/*z* dimensions ranked from 48/200/30 mm to 420/420/420 mm. The mean/range of sequence parameters is given in Table SM1.

In order to account for data origin bias, testing data originating from the two 3 T scanners, which were also used in the training phase (Ingenia and Achieva d-Stream, Philips Healthcare), was excluded in an additional analysis resulting in *n* = 66 remaining datasets. Respective results are provided in the supplementary material.

### Synthesis of sagittal T2-w fs images

**Fig. 3:**
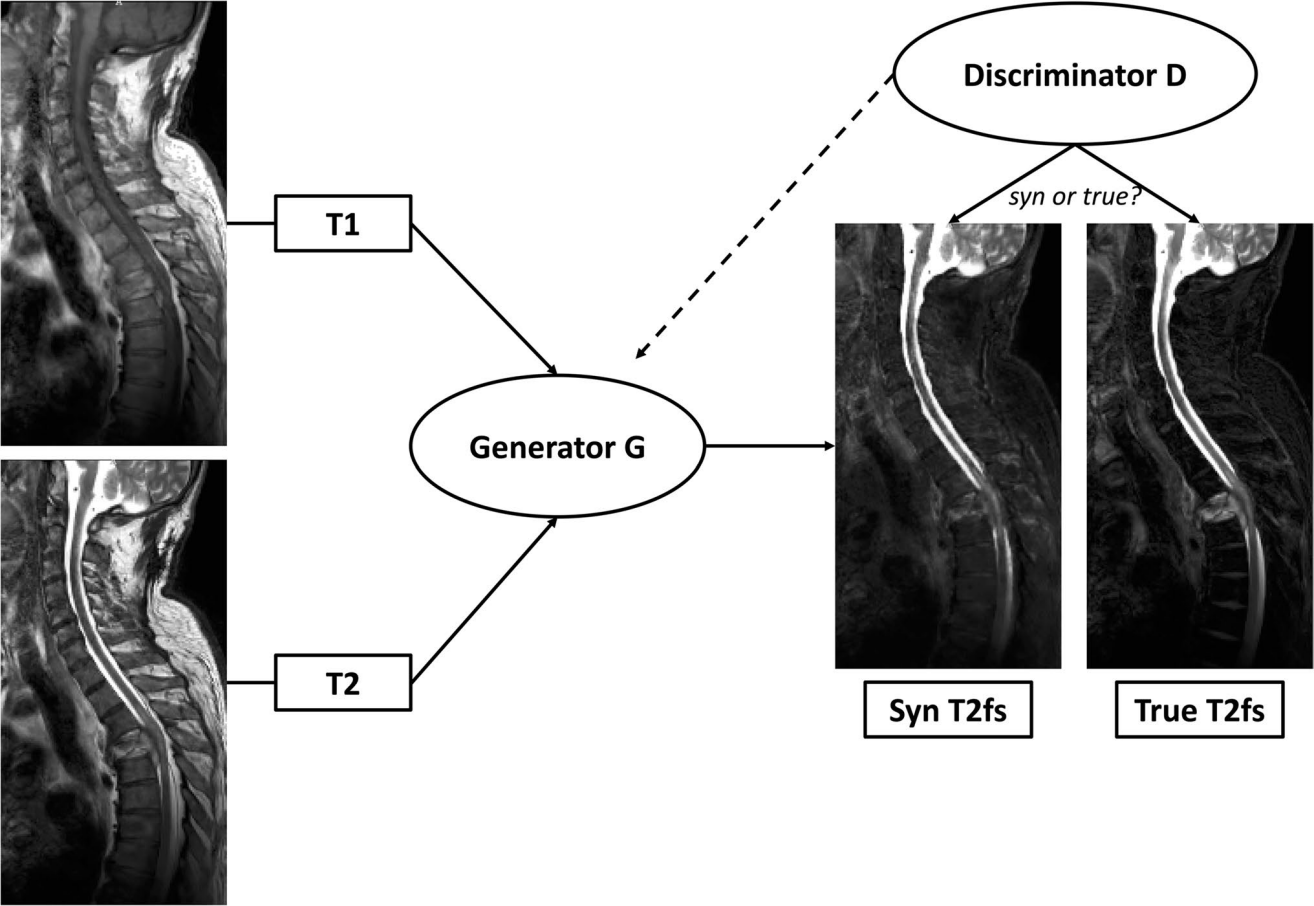
Diagram of architecture and training process of the synthesis task. The Generator G uses T1- and T2-w images to generate synthetic T2-w fs images. Feedback on the similarity between synthetic T2-w fs and true T2-w fs is offered by the Discriminator D and causes modifications in network weightings until the loss of function to discriminate between both images is minimal

The GAN for synthesis of sagittal T2-w fs images from T1-w and non-fs T2-w images is based on the pix2pix architecture by Isola et al. [30] (details are given in SM Appendix 1). The artificial generation of one T2-w fs dataset takes on average less than 5 min depending on the computational power. Most of this time is needed for image registration; the image synthesis by the GAN takes less than 30 s. A schematic diagram with exemplary images of the GAN architecture and the training process of image synthesis is shown in Fig. 3. The GAN model and one test case can be found in the following repository: https://doi.org/10.6084/m9.figshare.16627576

### Evaluation of GAN performance

#### Objective image quality evaluation

One neuroradiologist with six years of experience in spine imaging performed apparent signal- and contrast-to-noise ratio (aSNR/aCNR) measurements comparable to the work by Pennig et al. [31] in ten representative datasets of corresponding synthetic and true T2-w fs images (including internal and external data). A region of interest (ROI) was manually drawn in the same position on synthetic and true T2-w fs images in (i) a healthy-appearing vertebral body and (ii) a region of bone marrow abnormality. Additionally, a ROI was placed in the paraspinal muscles as a reference standard for background noise, assuming relatively homogenous muscle tissue and therefore relating signal standard deviation mainly to noise. The aSNR and aCNR were calculated as follows:

1 $$\mathrm{aSNR}=\frac{{\mathrm{SI}}_{\mathrm{healthy}\;\mathrm{vertebral}\;\mathrm{body}}}{{\mathrm{SD\ of\ SI}}_{\mathrm{muscle}}}$$

2 $$a\mathrm{CNR}=\frac{({\mathrm{SI}}_{\mathrm{bone}\;\mathrm{marrow}\;\mathrm{abnormality}}-{\mathrm{SI}}_{\mathrm{healthy}\;\mathrm{vertebral}\;\mathrm{body}})}{{\mathrm{SD\ of\ SI}}_{\mathrm{muscle}}}$$

where SI is the signal intensity, and SD is the standard deviation. For each dataset, aSNR and aCNR were calculated.

#### Subjective image and fat saturation quality evaluation

The 101 test datasets (T1-w, T2-w, synthetic T2-w fs, and true T2-w fs images) were investigated by two neuroradiologists (reader 1 with six years of experience; reader 2 with three years of experience in spine imaging). The expert readers blindly graded synthetic and true T2-w fs images regrading image quality based on a 5-point scale [16] and fat saturation quality based on a 3-point scale by assessing presence of artifacts, overall SNR, and image contrast (Table 1 (a)).

**Table 1:** Grading scores for image and fat saturation quality (a) and for the six different spine pathologies (b)

(a)	Grade					
	**1**	**2**	**3**	**4**	**5**	
**Image quality**	Poor	Marginal	Acceptable with moderate artifacts	Good with some artifacts	Excellent with minimal/no artifacts	
**Fat suppression/separation**	Weak	Medium	Good	–	–	
**(b) Pathologies**	Grade					
	**0**	**1**	**2**	**3**		**4**
**Bone marrow abnormalities**	Absent	Focal	One-third of vertebral body	Two-thirds of vertebral body		Whole vertebral body or affection of pedicles/proc. spinosus
**Spondylodiscitis expansion**	Absent	One-third of vertebral body	Two-thirds of vertebral body	Whole vertebral body		–
**Juxtadiscal Modic changes (inflammatory)**	Absent	Present	–	–		–
**Vertebral fractures**	Absent	Acute (edema present)	Chronic	–		–
**Cord lesions**	Absent	Present	–	–		–
**Paravertebral tissue abnormalities**	Absent	Inflammation	Hematoma	Other		–

To assess whether synthetic and true T2-w fs images are indistinguishable, a visual Turing test was performed. From the testing dataset 25 synthetic and 25 true T2-w fs images of the same patient, respectively, were presented randomized and blinded to eleven neuroradiologists (one to 20 years of experience in spine MRI) using a website-based graphical user interface (GUI) [32, 33]. Participants were obliged to classify the shown image as a synthetic or a true T2-w fs. Without learning whether the classification was correct or wrong, the subsequent image was presented.

#### Evaluation of diagnostic agreement

In each of the 101 test datasets, five consecutive vertebral segments were defined as ROI based on T1-w, T2-w, and true T2-w fs images. Thereby, throughout all datasets cervical, thoracic and lumbar spine segments were included. Subsequently, the two aforementioned expert readers assessed diagnostic agreement of the images by grading six different pathologies in the ROI: bone marrow abnormalities, spondylodiscitis expansion, Modic changes, vertebral fractures, spinal cord lesions, and paravertebral tissue abnormalities. The six named pathologies were chosen, as they are among the most common spinal pathologies. Particularly for these six pathologies, a sufficient fluid contrast is important for assessment and, therefore, the analysis of T2-w fs images is of significant diagnostic relevance. Grading scores are given in Table 1 (b). The two readers independently graded pathologies on the synthetic (T1-w, T2-w, and synthetic T2-w fs images) and the original protocol (T1-w, T2-w, and true T2-w fs images) in a randomized and blinded assessment.

#### Gold standard definition for accuracy

After completion of the blinded expert readings, a ground truth (GT) grading of the 101 test datasets was defined. T1-w, T2-w, and true T2-w fs images were assessed in a consensus grading of both expert readers, additionally incorporating the information of pre- or follow-up scans, other imaging modalities, and clinical information.

### Statistical analysis

Statistical analysis was performed with SPSS (version 27.0, IBM SPSS Statistics for MacOS, IBM Corp.) and Microsoft Excel (2021). A *p*-value of 0.05 was set as threshold for statistical significance.

Significant difference between aSNR and aCNR of synthetic and true T2-w fs images from the ten representative datasets was evaluated using the Wilcoxon signed-rank test.

Image and fat saturation quality grading of synthetic and true T2-w fs was analyzed using descriptive statistics. Significant differences between image and fat saturation quality grading of synthetic and true T2-w fs were evaluated using the Wilcoxon signed-rank test.

The Turing test was analyzed using descriptive statistics. Significant difference real condition versus expert grading between true and synthetic T2-w fs images was evaluated using McNemar’s test.

To evaluate the intermethod agreement of pathology assessment based on the synthetic versus the original protocol, Cohen’s kappa (*ĸ*) coefficients were calculated [34]. Also, the interrater agreement for pathology grading was calculated using Cohen’s *ĸ* coefficients. Significant differences between Cohen’s *ĸ* coefficients were evaluated using the Wilcoxon signed-rank test.

For comparison with the gold standard, accuracy of grading was calculated and corresponding significance was evaluated using a McNemar’s test.

## Results

### Image and fat saturation quality of synthetic versus true T2-w fs

aSNR and aCNR values for synthetic and true T2-w fs images of ten representative datasets were not significantly different (*p* > 0.05). The detailed results are provided in Table SM2a. For a comparison of objective and subjective image quality measures, Table SM2b provides corresponding image-quality grades of both expert readers for synthetic and true T2-w fs images, respectively.

The image quality of the synthetic T2-w fs was graded higher than that of the true T2-w fs by both readers (97.0% of synthetic T2-w fs images versus 87.6% of true T2-w fs images graded at least acceptable) (Table 2 (a)). The difference in image quality grading was statistically significant (*p* = 0.023). Quality of fat saturation grading was not significantly different between synthetic T2-w fs and true T2-w fs, with 84.7% of synthetic T2-w fs images and 81.7% of true T2-w fs images graded as good fat saturation (*p* > 0.05) (Table 2 (b)).

**Table 2:** Cross table image (a) and fat saturation (b) quality grading synthetic versus true T2-w fs for both readers. 1 indicates worst quality. In (a) significantly more cases favor synthetic images (bold italic, *n* = 67), than true T2-w fs images (italic; *n* = 49; *p* = 0.023). *n* = 86 cases in which image quality gradings of synthetic and true T2-w fs correspond

(a) Image quality	Synthetic T2-w fs					
True T2-w fs	**1 (poor)**	**2**	**3**	**4**	**5 (excellent)**	**Total**
1 (poor)	0	***0***	***0***	***1***	***1***	**2**
2	*0*	2	***6***	***12***	***3***	**23**
3	*0*	*2*	18	***18***	***9***	**47**
4	*0*	*2*	*13*	16	***17***	**48**
5 (excellent)	*0*	*0*	*10*	*22*	50	**82**
Total	**0**	**6**	**47**	**69**	**80**	**202**
(b) Fat saturation quality	**Synthetic T2-w fs**					
True T2-w fs	**1 (weak)**	**2**	**3 (good)**	**Total**		
1 (weak)	0	1	3	**4**		
2	4	8	21	**33**		
3 (good)	4	14	147	**165**		
Total	**8**	**23**	**171**	**202**		

Analysis of image and fat saturation quality of the remaining 66 datasets, when test data originating from the two scanners, that were also used in the training phase (Ingenia and Achieva d-Stream, Philips Healthcare) was excluded is provided in Table SM3.

**Fig. 4:**
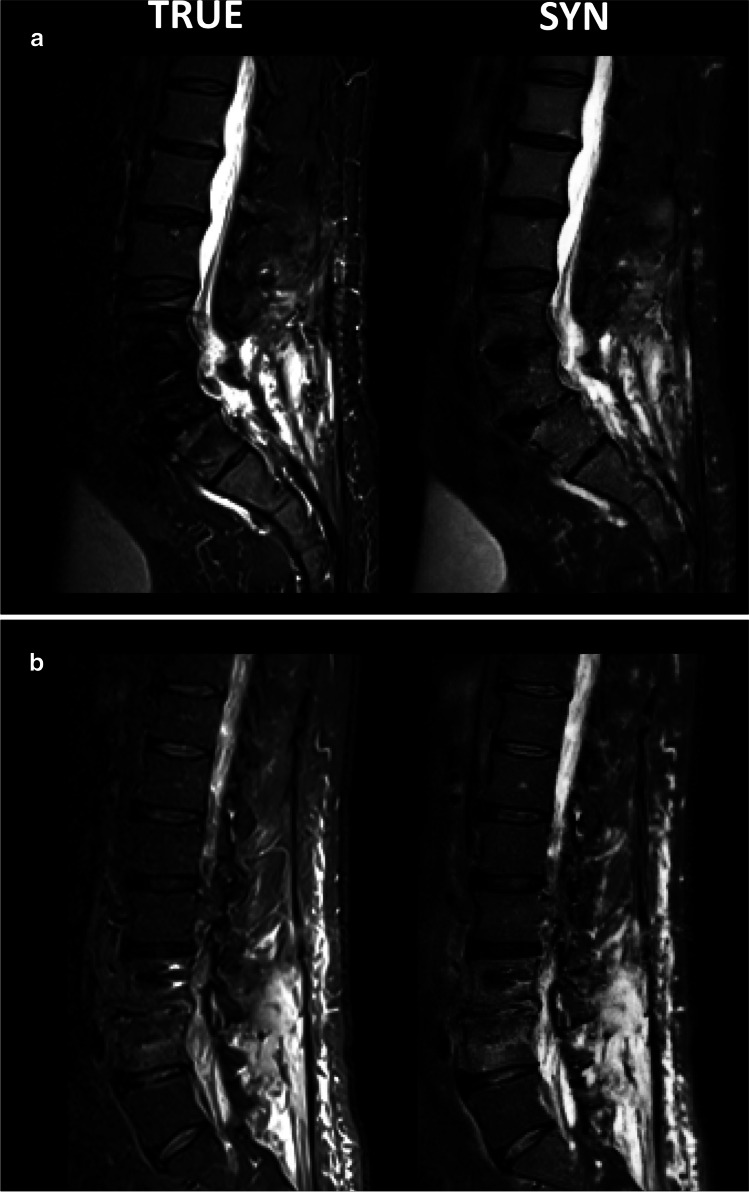
Representative true and synthetic T2-w fs images with metal implants (intervertebral disk cages and pedicle screws)

Visual inspection of cases with metal implants revealed a higher image quality in synthetic images. Figure 4 shows synthetic and true T2-w fs images with metal implants. The synthetic T2-w fs images were based on T1-w and T2-w sequences with specific metal artifact reduction techniques. Also, the true T2-w fs were sequences with metal artifact reduction. In both cases, the synthetic T2-w fs provided a better image quality than the true T2-w fs, offering a better SNR, higher contrast, and less artifacts surrounding the metal implants.

Based on the Turing test performed by eleven independent neuroradiologists, no significant difference real condition versus expert grading was observed between synthetic and true T2-w fs images (*p* > 0.05) (Table 3). 42.9% of synthetic T2-w fs images and 38.5% of true T2-w fs images were graded incorrectly as the respective counterpart.

**Table 3:** Cross-table visual Turing test condition (true/synthetic) versus grading (true/synthetic). Differences were not significant (*p* > 0.05)

	Condition		
Grading	True	Synthetic	Total
True	169	118	**287**
Synthetic	106	157	**263**
Total	**275**	**275**	**550**

### Diagnostic agreement between synthetic and original protocol

**Fig. 5:**
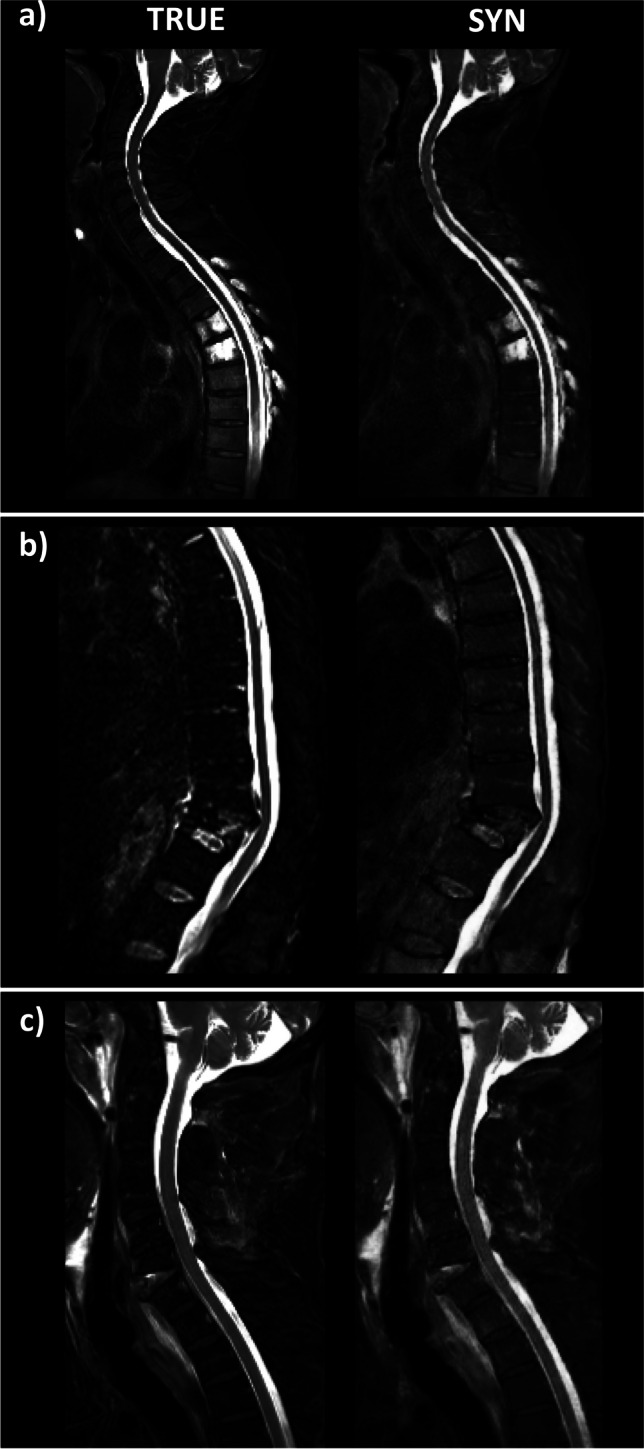
Representative true and synthetic T2-w fs images for different pathologies: **a** bone marrow abnormalities, **b** vertebral facture, and **c** paravertebral tissue abnormalities

Figure 5 shows representative synthetic and true T2-w fs images with bone marrow abnormalities, vertebral fractures, and paravertebral tissue abnormalities. The original images originate from different scanner vendors and field strengths. A purely qualitative visual comparison of the two juxtaposed images shows the similar diagnostic performance of synthetic versus true T2-w fs images regarding the detection of the presented spine pathologies.

Table 4 shows the intermethod agreement (Cohen’s *ĸ* coefficients) for grading based on the synthetic protocol compared with the original protocol for reader 1 and reader 2, respectively. For both readers, the intermethod agreement ranged from substantial to almost perfect agreement for all evaluated pathologies (bone marrow abnormalities, spondylodiscitis expansion, inflammatory Modic changes, vertebral fractures, cord lesions, and paravertebral tissue abnormalities), except for grading of spinal cord lesions by reader 1 which showed a moderate agreement. Cohen’s *ĸ* coefficients were significantly different between reader 1 and reader 2 (*p* = 0.046) (Table 4). The agreement between synthetic and original protocol by the same reader was higher than interrater agreement except for spinal cord lesions (Table 4, significance only found for reader 2, *p* = 0.028).

**Table 4:** Intermethod agreement (Cohen’s kappa coefficient) between synthetic protocol (T1-w, T2-w, and synthetic T2-w fs) and original protocol (T1-w, T2-w, and true T2-w fs) for reader 1 and 2; interrater agreement (Cohen’s kappa coefficient) for synthetic protocol and original protocol

	Intermethod Cohen's kappa		Interrater Cohen's kappa	
Pathology	Reader 1	Reader 2	Synthetic protocol	Original protocol
Bone marrow abnormalities	0.76	0.91	0.70	0.81
Spondylodiscitis expansion	0.85	0.91	0.74	0.59
Juxtadiscal Modic changes (inflammatory)	0.75	0.74	0.66	0.61
Vertebral fracture	0.78	0.91	0.80	0.81
Cord lesions	0.56	0.66	0.59	0.70
Paravertebral tissue abnormalities	0.79	0.86	0.74	0.77

Resulting Cohen’s *ĸ* coefficients of the remaining 66 datasets, when test data originating from the two scanners, that were also used in the training phase (Ingenia and Achieva d-Stream, Philips Healthcare) was excluded, are provided in Table SM4.

No significant difference between accuracy of synthetic and original protocol was shown ranging between 82.2% for grading of bone marrow abnormalities and 95.0% for grading of spondylodiscitis expansion (*p* > 0.05) (Table 5).

**Table 5:** Accuracy in % of grading based on the synthetic protocol and the original protocol, respectively. No significant difference was shown (*p* > 0.05)

Pathology	*n* (ground truth)	Accuracy synthetic protocol (%)	Accuracy original protocol (%)
Bone marrow abnormalities	61	82.2	82.7
Spondylodiscitis expansion	5	95.0	95.0
Juxtadiscal Modic changes (inflammatory)	28	87.1	85.1
Vertebral fracture	21	92.1	92.1
Cord lesions	15	90.0	93.6
Paravertebral tissue abnormalities	25	88.6	92.1

### Scan time reduction

In the validation dataset, acquisition duration of T1-w sequence was on average 155 s; of non-fs T2-w sequences, 207 s; and of T2-w fs sequences, 207 s. Waiving the physical acquisition of T2-w fs images consequently shortens the scan protocol by around 40% in a conventional spine examination.

## Discussion

Our work demonstrates the diagnostic potential of a GAN-based, sagittal T2-w fs in spine imaging. The synthetic T2-w fs images provided an overall better image quality than the true T2-w fs images, and pathology assessment on the synthetic protocol showed an excellent agreement with the original protocol. We could prove the generalizability of our approach as our assessment is based on a challenging, multicenter test dataset. Consequently, the synthetic T2-w fs might replace a physically acquired T2-w fs in the future, leading to a relevant reduction of scan time for pathology assessment in the spine.

With the introduction of DL techniques into the radiological workflow, synthetic MR contrasts based on GAN frameworks are emerging. Recently, feasibility studies demonstrated the clinical benefit of GAN-based MR images, e.g., a synthetic double inversion recovery (DIR) sequence improved lesion detection in multiple sclerosis [26]. Intrinsic MR contrasts such as T1 or T2 unlike gadolinium contrast can be synthesized without artifacts from other MR contrasts using GANs [21], potentially rendering the physical acquisition of particular MR sequences no longer necessary and thus reducing scan time.

Whereas objective image quality evaluation did not reveal significant differences between synthetic and true T2-w fs images, synthetic images showed a significantly better image quality than true T2-w fs images based on the grading by two expert readers. Our approach of virtually generating T2-w fs images with a GAN allows for an overall scan time reduction of around 40% in conventional spine examinations. This not only increases MR throughout, but might also be one reason for the significantly better image quality of synthetic T2-w fs images compared to true T2-w fs images. Due to reduced patient comfort during prolonged acquisition times and as the fs sequences are often acquired at the end, true T2-w fs images might be affected by motion artifacts. Additionally, MR fat saturation techniques are, depending on the used technique, prone to magnetic field inhomogeneities or inherently suffer from a lower SNR [4]. This is of particular concern, when regions with implanted hardware are scanned. In contrast, the T2-w fs generated by the GAN uses conventional T1-w and non-fs T2-w images as input, which are technically more stable, are less prone to artifacts, and offer higher SNR. Consequently, although it is known that artificially generated images using GANs can show particular artifacts [35], our synthetic T2-w fs images showed improved image quality.

Next to convincing image quality, synthetic images have to represent reality. Therefore, an excellent diagnostic agreement with the original protocol and high accuracy are of particular importance.

For five of the six evaluated pathologies, the expert grading based on the synthetic protocol (including the synthetic T2-w fs) showed a substantial to almost perfect agreement with the original protocol (including the true T2-w fs images). The assessment of spinal cord lesions by reader 1 merely showed a moderate agreement between the synthetic and the original protocol. Remarkably also, the interrater Cohen’s *ĸ* coefficient for evaluation of cord lesions based on the synthetic protocol is lower than the other interrater Cohen’s *ĸ* coefficients. Two aspects might explain the lower Cohen’s *ĸ* coefficients for grading of cord lesion: (1) The GAN was trained exclusively on T2-w Dixon fs images. However, particularly for the detection of cord lesions, T2-w short tau inversion recovery (STIR) images are recommended, whereas the Dixon fs technique is not considered ideal [12]. (2) Hyperintensities on T2-w fs images characterizing cord lesions on sagittal images are often subtle and inconclusive. Additional axial imaging can be helpful to distinguish hyperintensities on T2-w fs images from artifacts and to detect small, marginally located lesions [12]. Such sequences were not available here.

The excellent accuracy of expert grading based on the synthetic as well as on the original protocol, which showed no significant difference, underlines the good agreement of pathology assessment on synthetic images with the gold standard.

For a clinical implementation of GAN-based synthetic images, external validity is required. To the best of the authors’ knowledge, to date, the only two publications presenting GAN-based T2-w fs images in the spine employed MR images from one single vendor [15, 29]. In our work, the GAN framework has been tested on multicenter data. The 101 testing datasets consisting of T1-w and non-fs T2-w images originated from 38 different scanners, with 41 datasets from 1.5 T and 60 datasets from 3 T systems. In contrast to previous studies in brain and spine datasets with a homogeneous FOV, our study demonstrated that GANs can reliably be applied in cases with a highly variable FOV. We were able to demonstrate the generalizability of our approach, by training the network with images from two scanners only and validating it on unseen images derived from 38 different scanners of various field strengths, acquisition protocols, and manufacturers.

The present study has limitations. First, the higher image quality of synthetic compared to true T2-w fs images might lead to bias, when in the course of the grading procedure readers are learning to notice subtle intrinsic image features allowing a differentiation in few samples. In order to rule out a relevant learning bias, we additionally performed a visual Turing test. By randomly presenting synthetic and true T2-w fs images to a broad annotator group without giving feedback about mistakes [36], we could prove that synthetic and true T2-w fs images cannot be significantly distinguished from each other.

Second, the two expert readers had slightly different clinical experience, which might account for some interrater variability.

Third, in our study, only sagittal images have been assessed, although in the clinical routine potentially axial and coronal images are part of spine MRI examinations [37]. However, all current imaging protocol recommendations do not include axial fs images in their recommendations [2]. Sagittal images are often used as screening images to guide the exact ROI for (non-fs) axial imaging. As consequently, sagittal imaging plays a major role in radiological spine assessment, the present study is meant to concentrate on sagittal images.

Fourth, for the proposal of a new technique in the clinical setting, a power analysis is necessary. However, to perform a power analysis, we need some initial information of the performance and suspected diagnostic value of such a new technique that was not available prior to our work presented here. Our study is meant to preliminarily analyze the general potential of GAN-based, synthetic T2-w fs images of the spine and shows the non-inferiority of synthetic T2-w fs images compared to true T2-w fs images in a heterogenous testing datasets. Further research with a power analysis simulating the routine radiological workflow is necessary to assess the additional diagnostic value of synthetic images particularly in the clinical setting.

## Conclusion

Our work underlines the potential of a GAN-based T2-w fs for scan time reduction in spine imaging. The overall better image quality and the excellent intermethod agreement render the synthetic T2-w fs a good alternative compared to the true T2-w fs. Our approach is highly generalizable as the assessment is based on a challenging, multicenter test dataset. Therefore, our GAN-based T2-w fs might replace a physically acquired T2-w fs in the future.

## Supplementary Information

Below is the link to the electronic supplementary material.

Supplementary file1 (DOCX 35.3 kb)

## Abbreviations and acronyms

aCNR: Apparent contrast-to-noise ratio
DIR: Double inversion recovery
DL: Deep learning
fs: Fat sat
FOV: Field of view
GAN: Generative adversarial network
GT: Ground truth
GUI: Graphical user interface
*ĸ*: Kappa
MRI: Magnetic resonance imaging
ROI: Region of interest
STIR: Short tau inversion recovery
aSNR: apparent signal-to-noise ratio
TSE: Turbo spin echo
w: Weighted

## Declarations

### Guarantor

The scientific guarantor of this publication is Jan S. Kirschke.

### Statistics and biometry

PD Dr. Alexander Hapfelmeier (Dipl.-Stat.) (Institute of General Practice and Health Services Research and Institute of Medical Informatics, Statistics and Epidemiology, Technical University of Munich, Munich, Germany) kindly provided statistical advice for this manuscript.

### Methodology

• retrospective

• diagnostic or prognostic study

• performed at one institute with multicenter data
