# Supplementary material for: Synthetic T2-weighted fat sat based on a generative adversarial network shows potential for scan time reduction in spine imaging in a multicenter test dataset
Source: Eur Radiol. 2023 Mar 16;33(8):5882–93. doi: 10.1007/s00330-023-09512-4 (PMC10326102; doi:10.1007/s00330-023-09512-4)
Supplement: Supplementary file 1 — Supplementary file1 (DOCX 35.3 kb) [file 330_2023_9512_MOESM1_ESM.docx]

**Supplementary Material:**

**SM Appendix 1:**

T1-w, T2-w and T2-w fs images were linearly co-registered using ANTs and resampled to 1x1 mm in-plain resolution. The images were winsorized between the 1^st^ and 99^th^ percentile and normalized into a [-1;1] range.

The GAN for synthesis of sagittal T2-w fs images is based on the pix2pix architecture introduced by Isola et al. (Isola P. IEEE Conference on Computer Vision and Pattern Recognition (CVPR), 2017). The generator in our network is a standard U-Net (Ronneberger O. Cham: Springer International Publishing, 2015) with the addition of dropout layers in the decoder part, the discriminator is patch-based (Ledig C. IEEE Conference on Computer Vision and Pattern Recognition (CVPR), 2017). While the discriminator D learns to differentiate between true and synthetic T2-w fs images (conditional on the input images) and is therefore driven by a binary cross-entropy (BCE) loss, the generator G is optimizing a joint loss:

Loss_G_ = (1-SSIM(true,synthetic)) + (λ * BCE(1,D(synthetic)))

Here, SSIM is the structural similarity index measure, a metric capturing the similarity between two images (Wang Z. IEEE Trans. Image Process, 2004). To enforce the generator to create “realistic” images, the loss also includes the discriminator’s “judgement” on the synthetic image. λ is a hyperparameter balancing the two loss components and was empirically set to 50 in our study.

Training was done slice-wise (sagittal slices), with spatial (flipping, rotation) and intensity (gaussian smoothing, random noise) augmentations. As is standard, the discriminator and the generator were trained in turns, using the Adam optimizer with a learning rate of 2e-4. Training was run for 25 epochs, where one epoch represented one loop over all training slices (in random order for each epoch).

**Table SM1: Sequence parameters of GAN training dataset and testing dataset.**

|  | **Training data**  2 internal scanners | | | **Test data** (mean; range)  5 internal scanners and 33 external scanners | | |
| --- | --- | --- | --- | --- | --- | --- |
|  | **T1-w** | **non-fs T2-w** | **fs T2-w** | **T1-w** | **non-fs T2-w** | **fs T2-w** |
| **TR [ms]** | 494 | 2517 | 2517 | 603; 13 -960 | 3176; 2400 - 6600 | 3369; 2500 - 6630 |
| **TE [ms]** | 8 | 100 | 100 | 11; 5 - 57 | 102; 80 - 126 | 84; 35 - 270 |
| **Slice gap [mm]** | 3.3 | 3.3 | 3.3 | 3.4; 2.2 - 5.4 | 3.5; 2.2 - 5.4 | 3.4; 2.2 - 5.5 |
| **Averages** | 1 | 1 | 1 | 1.5; 1 - 5 | 1.3; 1 - 3 | 1.5; 1 - 3 |
| **FOV xdim [mm]** | 280 | 280 | 280 | 291; 48 - 420 | 291; 200 - 420 | 293; 54 - 420 |
| **FOV ydim [mm]** | 280 | 280 | 280 | 293; 200 - 420 | 292; 200 - 420 | 302; 200 - 420 |
| **FOV zdim [mm]** | 56 | 56 | 56 | 55; 30 - 256 | 55; 33 - 420 | 64; 33 - 320 |
| **Scan duration [s]** | 154 | 186 | 186 | 155; 100 - 283 | 207; 102 - 349 | 207; 133 - 349 |

**Table SM2: Apparent signal- and contrast-to-noise-ratio (aSNR/aCNR) for synthetic and true T2-w fs images of ten representative datasets. Values were not significantly different between synthetic and true images (*p* > 0.05) (a). Subjective image quality grades of reader 1 and 2 for synthetic und true T2-w fs images for the ten representative datasets in which aSNR and aCNR ratio were calculated (b).**

**(a)**

| **Subject Nr.** | **aSNR  Synthetic T2-w fs** | **aSNR  True T2-w fs** | **aCNR**  **Synthetic T2-w fs** | **aCNR**  **True T2-w fs** |
| --- | --- | --- | --- | --- |
| **01** | -27.22 | -24.92 | 51.90 | 65.80 |
| **02** | -20.72 | -22.57 | 8.16 | 4.09 |
| **03** | -12.33 | -0.41 | 17.97 | 9.54 |
| **04** | -17.59 | -19.58 | 13.61 | 37.84 |
| **05** | -13.06 | -32.28 | 23.64 | 73.77 |
| **06** | -6.35 | -2.07 | 6.92 | 0.98 |
| **07** | -18.59 | -13.74 | 11.10 | 13.32 |
| **08** | -10.93 | -13.95 | 24.22 | 43.30 |
| **09** | -9.92 | 0.22 | 6.93 | 6.02 |
| **10** | -25.09 | -12.73 | 10.04 | 31.45 |

**(b)**

| **Subject Nr.** | **Image Quality Reader 1**  **Synthetic T2-w fs** | **Image Quality Reader 2**  **Synthetic T2-w fs** | **Image Quality Reader 1**  **True T2-w fs** | **Image Quality**  **Reader 2**  **True T2-w fs** |
| --- | --- | --- | --- | --- |
| **01** | 5 | 4 | 5 | 4 |
| **02** | 3 | 4 | 4 | 5 |
| **03** | 5 | 5 | 5 | 5 |
| **04** | 4 | 3 | 3 | 3 |
| **05** | 4 | 4 | 2 | 2 |
| **06** | 3 | 3 | 4 | 4 |
| **07** | 5 | 3 | 5 | 4 |
| **08** | 4 | 3 | 5 | 4 |
| **09** | 4 | 4 | 3 | 3 |
| **10** | 4 | 3 | 4 | 5 |

**Table SM3: Cross table image (a) and fat saturation (b) quality grading synthetic versus true T2-w fs for both readers (in total n = 132 cases), when data from the two scanners, that were also used in the training phase (Ingenia and Achieva dStream), was excluded. 1 indicates worst quality. In (a) significantly more cases favor synthetic images (bold italic, n = 61), than true T2-w fs images (italic; n = 20; *p* < 0.001). n = 51 cases in which image quality gradings of synthetic and true T2-w fs correspond. Quality of fs grading was not significantly different between synthetic T2-w fs and true T2-w fs (*p* > 0.05).**

**(a)**

| **Image quality** | **Synthetic T2-w fs** | | | | | |
| --- | --- | --- | --- | --- | --- | --- |
| **True T2-w fs** | **1 (poor)** | **2** | **3** | **4** | **5 (excellent)** | **Total** |
| **1 (poor)** | 0 | ***0*** | ***0*** | ***1*** | ***1*** | **2** |
| **2** | *0* | 1 | ***4*** | ***12*** | ***3*** | **20** |
| **3** | *0* | *0* | 9 | ***15*** | ***8*** | **32** |
| **4** | *0* | *0* | *3* | 11 | ***17*** | **31** |
| **5 (excellent)** | *0* | *0* | *5* | *12* | 30 | **47** |
| **Total** | **0** | **1** | **21** | **51** | **59** | **132** |

**(b)**

| **Fat saturation quality** | **Synthetic T2-w fs** | | | |
| --- | --- | --- | --- | --- |
| **True T2-w fs** | **1 (weak)** | **2** | **3 (good)** | **Total** |
| **1 (weak)** | 0 | 0 | 3 | **3** |
| **2** | 3 | 6 | 19 | **28** |
| **3 (good)** | 2 | 8 | 91 | **101** |
| **Total** | **5** | **14** | **113** | **132** |

**Table SM4: Intermethod agreement (Cohen’s Kappa coefficient) between synthetic protocol (T1-w, T2-w, and synthetic T2-w fs) and original protocol (T1-w, T2-w, and true T2-w fs) for reader 1 and 2 and interrater agreement (Cohen’s Kappa coefficient) for synthetic protocol and original protocol. In this table, data from the two scanners, that were also used in the training phase (Ingenia and Achieva dStream), was excluded (remaining cases n = 66).**

**No statistically significant differences between the calculated Cohen’s Kappa coefficients in this table and Cohen’s Kappa coefficients in Table 5 (including data from the two scanners of the training phase) were found (Wilcoxon signed-rank test: *p* > 0.05).**

|  | **Intermethod Cohen’s Kappa** | | **Interrater Cohen’s Kappa** | |
| --- | --- | --- | --- | --- |
| **Pathology** | **Reader 1** | **Reader 2** | **Synthetic Protocol** | **Original Protocol** |
| Bone marrow abnormalities | **0.77** | **0.89** | **0.68** | **0.77** |
| Spondylodiscitis expansion | **0.79** | **1.00** | **0.65** | **0.55** |
| Juxtadiscal Modic changes (inflammatory) | **0.75** | **0.81** | **0.61** | **0.64** |
| Vertebral fracture | **0.80** | **0.95** | **0.82** | **0.80** |
| Cord lesions | **0.53** | **0.58** | **0.45** | **0.72** |
| Paravertebral tissue abnormalities | **0.78** | **0.86** | **0.79** | **0.76** |
